# Outcomes at 7 Years of Age of Former Very Preterm Neonates with Repeated Surfactant Treatment for Prolonged Respiratory Distress in the Neonatal Period

**DOI:** 10.3390/jcm12196220

**Published:** 2023-09-27

**Authors:** Jean-Michel Hascoet, Hélène Deforge, Silvia Demoulin, Jean-Charles Picaud, Veronique Zupan, Isabelle Ligi, François Moreau, Aurelie Labarre, Patrick Daoud, Laurent Storme, Claude Bonabel, Isabelle Hamon

**Affiliations:** 1 DevAH Research Unit, Lorraine University, 54500 Vandoeuvre, France; h.deforge@chru-nancy.fr (H.D.); silvia.demoulin@chu-lille.fr (S.D.); i.hamon@chru-nancy.fr (I.H.); 2 Maternite Regionale, Service de Médecine et Réanimation Néonatale, Centre Hospitalier Régional Universitaire, 54035 Nancy, France; 3 Service Exploration Fonctionelle Respiratoire, Centre Hospitalier Universitaire, 54500 Vandoeuvre, France; c.bonabel@gmail.com; 4 Service de Néonatologie et de Réanimation Néonatale, Hospices Civils de Lyon, Hôpital de la Croix Rousse, 69004 Lyon, France; jean-charles.picaud@chu-lyon.fr; 5 Service de Réanimation Néonatale, Hôpital A. Béclère, 92141 Clamart, France; veronique.zupan@aphp.fr; 6 Service de Médecine et Réanimation Néonatale, Centre Hospitalier Universitaire La Conception, 13385 Marseille, France; isabelle.ligi@ap-hm.fr; 7 Service de Médecine Néonatale, Surveillance Continue et Réanimation Pédiatrique Polyvalente, Centre Hospitalier Universitaire Amiens-Picardie, 80000 Amiens, France; moreau.francois@chu-amiens.fr; 8 Unité de Réanimation Pédiatrique et Néonatale, Centre Hospitalier Universitaire Charles Nicolle, 76000 Rouen, France; aurelie.labarre@chu-rouen.fr; 9 Département Femmes/Enfants, Centre Hospitalier Intercommunal A Grégoire, 93100 Montreuil, France; patrick.daoud@ght-gpne.fr; 10 Secteur Réanimation Néonatale, Service de Médecine Néonatale, Centre Hospitalier Régional Universitaire, 59037 Lille, France; laurent.storme@chu-lille.fr

**Keywords:** extreme prematurity, surfactant, outcome, children, longitudinal study, pulmonary function testing, neurodevelopment, asthma

## Abstract

This study aimed at evaluating the 7-year outcomes of 118 very preterm newborns (VPNs, gestational age = 26 ± 1.4 w) involved in a randomized controlled trial. They presented neonatal respiratory distress (RDS), requiring ventilation for 14 ± 2 days post-natal age (PNA). A repeated instillation of 200 mg/kg poractant alfa (SURF) did not improve early bronchopulmonary dysplasia, but the SURF infants needed less re-hospitalization than the controls for respiratory problems at 1- and 2-year PNA. There was no growth difference at 7.1 ± 0.3 years between 41 SURF infants and 36 controls (80% of the eligible children), and 7.9% SURF infants vs. 28.6% controls presented asthma (*p* = 0.021). The children underwent cognitive assessment (WISC IV) and pulmonary function testing (PFT), measuring their spirometry, lung volume, and airway resistance. The spirometry measures showed differences (*p* < 0.05) between the SURF infants and the controls (mean ± standard deviation (median z-score)) for FEV1 (L/s) (1.188 ± 0.690(−0.803) vs. 1.080 ± 0.243 (−1.446)); FEV1 after betamimetics (1.244 ± 0.183(−0.525) vs. 1.091 ± 0.20(−1.342)); FVC (L) (1.402 ± 0.217 (−0.406) vs. 1.265 ± 0.267 (−1.141)), and FVC after betamimetics (1.452 ± 0.237 (−0.241) vs. 1.279 ± 0.264 (−1.020)). PFT showed no differences in the volumes or airway resistance. The global IQ median (interquartile range) was 89 (82:99) vs. 89 (76:98), with 61% of the children >85 in both groups. Repeated surfactant treatment in VPNs presenting severe RDS led to the attenuation of early lung injuries, with an impact on long-term pulmonary sequelae, without differences in neurodevelopmental outcomes.

## 1. Introduction

Advances in perinatal care have made it possible to significantly improve the survival of most immature neonates. However, this improvement accounts for some of the increase in bronchopulmonary dysplasia (BPD) [1,2]. Indeed, despite good obstetric practice and regionalization [3], prenatal maturation by maternal corticosteroids [4,5] and the early use of surfactant replacement therapy [6,7], the improvement in neonatal survival is not associated with a lower risk of developing severe BPD, contributing to long-term morbidity, such as chronic lung disease, asthma, impaired neurodevelopment, and increased resource utilization [8,9]. BPD is a perinatal disease caused by multiple factors, for which diverse therapeutic approaches have been proposed [3,5,6,10,11]. These measures have somewhat limited the development of BPD but are not enough effective or devoid of significant side effects in the long term.

Among the most effective measures, exogenous surfactant administration has become a standard of care for very premature neonates [7]. The modalities of optimal surfactant administration at birth are now well defined [3,7], with early instillation after continuous positive airway pressure from birth, and an early second dose in case of persistent respiratory distress (RDS) [12]. The most recent approaches using minimizing invasive ventilation without intubation allow for lowering the risk associated with mechanical ventilation sequelae [6]. However, some very premature infants still present severe RDS, with prolonged mechanical ventilation and high oxygen requirements. Repeated exogenous surfactant treatment in these neonates, presenting prolonged pulmonary disease depending on the respirator and risking progression to severe BPD, should theoretically allow for promoting pulmonary healing and growth [10,11,13,14]. Thus, we designed a double-blind, randomized, multicentric controlled trial to evaluate the effects of one late repeated surfactant administration in very preterm infants of less than 33 weeks of gestation, who still required mechanical ventilation at 14 days of age [15]. In this study, we observed that the 118 newborns involved presented a very high rate of associated infection. After surfactant instillation but not air, the FiO2 requirements dropped for up to 24 h after instillation, but there was only a trend for less severe bronchopulmonary dysplasia/death rates at 36 weeks of postmenstrual age (PMA) (27.1% vs. 35.6%; *p* = 0.32). Nevertheless, significantly fewer surfactant-treated infants needed re-hospitalization for respiratory problems within the first year after discharge in 91 of the 96 surviving infants (28.3% vs. 51.1%; *p* = 0.03). Likewise, at two years of age, the children hospitalized during their first year of life were more likely to be re-hospitalized during their second year (OR 4.182, 95% CI 1.478–11.834) [16]. The aims of this preplanned longitudinal study were to evaluate the pulmonary and neurodevelopment outcomes at 7 years of age of the infants involved in the multicenter controlled randomized trial in the neonatal period.

## 2. Materials and Methods

All surviving infants of the CURDYS trial [15] were eligible for this pre-planned 7-year longitudinal study. The investigating centers contacted the families by letter reminding them of the 7-year follow-up visit and inviting them to call the units to make an appointment. In the event of no response, in accordance with the previously signed agreement, they were called by telephone.

### 2.1. Parent Interview and Questionnaire

The parents were first interviewed about the events that had occurred since the 2-year visit. Then, they were prompted to respond to an auto-questionnaire evaluating their feelings on the impact of respiratory symptoms on their children and their family’s quality of life. This questionnaire was translated and adapted for the study from Powell et al.’s questionnaire, with 31 questions rated from 0 (no impact) to 4 (severe impact) [17]. It evaluated 4 domains, leading to a global score: diurnal symptoms, night symptoms, impact on child’s quality of life, and impact on family’s quality of life. The parents’ questionnaire specifically asked the question: “Has a doctor ever diagnosed your child with asthma? What medications did he prescribe for that problem?” In addition, at the time of the clinical visit, the pediatrician verified and recorded the diagnosis of asthma in the child’s health record with the treatment given. Both an indication of asthma had to be present in the questionnaire and the child’s health record to be recorded as “asthma”. The parents’ education levels were recorded and stratified into 4 groups: elementary or primary school, high school, baccalaureate degree, and college degree achievement. The children underwent a complete physical examination by certified trained pediatricians who were not aware of the group they had been randomized to. The standardized physical examination recorded each child’s weight, height, head circumference, heart rate, and resting arterial blood pressure. The children also had a complete clinical neuro-motor examination corresponding to their age.

### 2.2. Pulmonary Function Testing

Pulmonary function testing (PFT) of the children was performed using Jaeger MasterScreen PFT and Body System (CareFusion, Höchberg, Germany) by certified examiners who were also unaware of the children’s original conditions and blinded to their group allocation. Lung function tests were performed according to the American Thoracic Society/European Respiratory Society standards [18]. For the spirometry, we used an electronic spirometer, allowing for the real-time visualization of the flow–volume loop (SPIRO-USB EOLYS^®^, SPCS2.4, 69160 Tassin, France). The necessary respiratory maneuvers were explained to the children, who learned to perform forced acceleration using instructional software (Spirometry PC Software, MD Spiro^®^ Anaheim, CA, USA). After learning this, the children performed at least three acceptable maneuvers according to the ERS/ATS guidelines. Examining the data from all of the usable flow–volume curves enabled us to select the largest forced vital capacity (FVC, L), the forced expiratory volume in the first second (FEV1, L/s), and the maximum mid-expiratory flow values (MMEF, L/s). In order to assess the change in respiratory function in response to bronchodilator administration, bronchodilator responsiveness testing was also performed using four separated doses of 100 μg of salbutamol delivered in 30 s intervals, and at least three additional acceptable spirometry maneuvers were recorded 10–15 min after the betamimetics (β+) administration. The static lung volume, measured by the method of dilution of helium in a closed circuit, determined the functional residual capacity (FRC, L) total lung capacity (TLC, L), and vital capacity (VC, L). Finally, the children had to simply inhale calmly through a mouthpiece to measure the pressure and flow rate signals, allowing for the calculation of respiratory impedance from which we extracted the airway resistance, as determined by the loss of load in the phase with the flow rate (sRaw, cmH20/L.s).

### 2.3. Neurodevelopmental Evaluation

Finally, the Wechsler Intelligence Scale for Children, 4th edition (WISC-IV) [19], the latest version, was used to evaluate the children’s neuropsychological and cognitive functions. Four indices were combined to determine the abbreviated representative intelligence quotient of the children: similarities for the Verbal Comprehension Index, matrices reasoning for the Perceptual Reasoning Index, letter/number sequences for the Working Memory Index, and timed symbol search for the Processing Speed Index.

In the similarities test (Verbal Comprehension Index), which assesses abstraction, conceptualization, and categorization abilities, the children had to report the link between two words (elbow/knee; ice/steam, etc.). In the matrices reasoning test (Perceptual Reasoning Index), they had to choose, among several answers, the image completing a matrix by analogy. In the letter/number sequences test (Working Memory Index), following the utterance of a group of letters and “mixed” numbers, the children were instructed to recall the numbers in ascending order and then the letters in alphabetical order. This test assesses the ability to memorize and manipulate information in working memory. In the timed symbols test (Processing Speed Index), the children had to indicate, on several format pages and for two minutes, whether they found an isolated “target” symbol among a series of three symbols. This test assesses the speed of perception, processing, and response. The completion time for these four tests was approximately thirty minutes. The expected normal values were 10 ± 3.

### 2.4. Statistics

The descriptive data are presented as the mean ± standard deviation (SD) and the median values with the interquartile range (IQR). Comparisons between the groups were performed using a Chi-square test or Fisher’s exact test for the categorical variables when appropriate, and the Mann–Whitney U test or the Kruskal–Wallis with the Dwass–Steel–Critchlow–Fligner test for pairwise comparisons of the continuous variables. The results of the spirometry measurements are expressed as the mean ± SD and z-score, using the Global Lung Function Initiative reference (GLI-12) [20]. For all analyses, the alpha risk was set at 0.05, and the tests were two-sided. The statistical analyses were performed using SYSTAT 13 software (2009; Systat Software Inc., San Jose, CA, USA).

## 3. Results

### 3.1. Patients and Health Resource Utilization

From the 118 infants initially randomized by the 13 centers involved, 11 died in each group in the neonatal period. We were able to evaluate 77 out of the 96 surviving eligible infants (80%), with 41 SURF infants and 36 controls. Table 1 presents the characteristics of the population. There were no differences between the SURF infants and the controls, respectively, for passive smoking exposure (37 vs. 45%, *p* = 0.484), the number of infants with at least one hospitalization since the 2-year visit (41.4 vs. 41.7%, *p* = 0.986), the use of physiotherapy for respiratory purposes (14.6 vs. 25.0%, *p* = 0.252), or motricity (14.6 vs. 19.4%, *p* = 0.574).

### 3.2. Parent Auto-Questionnaire

The same proportions of parents agreed to respond to the auto-questionnaire (38/41 and 33/36 parents of the SURF infants and the controls, respectively). Table 2 presents the parents’ responses to the questionnaire. The parents reported that 7.9% of the SURF infants vs. 28.6% of the controls (*p* = 0.021) were diagnosed with asthma and presented symptoms requiring treatment.

### 3.3. Pulmonary Function Testing

A total of 57 children (74%) underwent complete pulmonary function testing: 26 SURF and 31 controls. However, two SURF infants and one control child were not able to perform the tests, and three SURF infants were not able to understand the instructions. Table 3 presents the results of the evaluated children.

In addition to the comparison between the two groups at 7 years of age, there were significant differences (*p* < 0.05) between the children who were not hospitalized at 2 years in the SURF infants (n = 20) and those who had to be hospitalized (n = 6), and in the non-hospitalized controls (n = 22) and those who had to be hospitalized (n = 9), respectively, for FEV1 (1.202 ± 0.161 vs. 1.112 ± 0.208 for the SURF infants, and 1.124 ± 0.244 vs. 0.903 ± 0.144 for the controls), and after β+ (1.293 ± 0.157 vs. 1.100 ± 0.197, and 1.139 ± 0.208 vs. 0.944 ± 0.140), for FVC (1.425 ± 0.201 vs. 1.308 ± 0.297, and 1.314 ± 0.264 vs. 1.080 ± 0.188), the FVC z-score (−0.344 ± 1.000 vs. −0.796 ± 1.650, and −0.940 ± 1.191 vs.−1.946 ± 1.047) and after β+ (1.509 ± 0.232 vs. 1.283 ± 0.175, and 1.335 ± 0.270 vs. 1.112 ± 0.171), and also for the vital capacity (1.407 ± 0.181 vs. 1.364 ± 0.301, and 1.335 ± 0.259 vs. 0.972 ± 0.271).

### 3.4. Neuro-Psychological and Cognitive Evaluation

The majority of the evaluated children were in their normal school grade (87% and 94% of SURF infants and controls, respectively) but 63 vs. 44% (*p* = 0.095) required specialized assistance at school. There were no differences in the use of speech therapy (53.7 vs. 66.7%, *p* = 0.246), the need for psychotherapy support (43.0 vs. 33.3%, *p* = 0.343), or recourse to a specialized institution (36.5 vs. 30.6%, *p* = 0.577) in the SURF and the control children, respectively.

We were able to obtain the data for the four selected subtests, one for each scale, in order to determine the abbreviated representative IQ (aIQ) in 64 children (33 vs. 31). Unfortunately, three SURF and four control children were not able to pass the WISC-IV evaluation. Other children did not show up for their neuropsychological evaluation. There were no significant differences between the two groups in any subtest (Table 4). Overall, for the aIQ evaluation, 61% of the children were above 85 in both groups, 18 vs. 19% were between 85 and 70, and 21 vs 20% were below 70 for the SURF vs. the control children, respectively.

## 4. Discussion

This pre-planned longitudinal study shows that former very preterm neonates presenting prolonged severe neonatal RDS and treated with one repeated surfactant dose at 14 days of age had improved lung function with less obstructive indicators, and presented fewer asthma symptoms at 7 years post-natal age. This is in line with the respiratory follow-up at 1 [15] and 2 years [16] of age, which showed significantly lower health resource utilization and less of a need for re-hospitalization for respiratory problems in the children who had received one repeated instillation of 200 mg/kg poractant alfa in the neonatal period, as compared to the control infants. The parents’ questionnaires evaluating their feelings on the impact of respiratory symptoms on the children and the family’s quality of life showed similar rather good tolerance in both groups. Finally, this study shows that although there were no significant differences between the two groups in the clinical, neurodevelopment, or cognitive outcomes, about 20% of the surviving children may be regarded as neurologically severely impaired.

These results are consistent with the clinical status of the infants at discharge, suggesting a difference in the severity of morbidity at the end of the neonatal hospitalization. Surfactant-treated neonates had significantly less necrotizing enterocolitis and earlier full enteral feeding and were discharged about 1 week earlier than the controls [15]. In a study of 165 extremely low-birth-weight neonates, Katz et al. evaluated 25 infants (representing 20% of the infants with RDS in their study) who received a repeated course of surfactant therapy for severe persistent respiratory failure and surfactant slump. They found that 70% of them had significant improvement in their lung disease up to 48 h after instillation [21], but no longer follow-up was evaluated. In a previous study, we showed that 42 VPNs compared to 27 healthy term-born children—non-asthmatic and non-atopic—had significant bronchoconstriction indicators of PFT at 7 years of age [22]. In addition, prematurity, chronic lung disease, the duration of mechanical ventilation or oxygen exposure, and maternal smoking were significant determinants of exercise-induced bronchoconstriction [22]. In the actual study, there were not enough children to validly evaluate the impact of hospitalization at 2 years of age between the two groups. However, within each group, the children who required hospitalization in the first 2 years of life also exhibited poorer lung function at 7 years of age than the infants who did not require re-hospitalization after discharge from the NICU. This suggests that re-hospitalization within the first 2 years of life may be an early predictor of chronic lung injury leading to long-term respiratory sequelae. In a review of the respiratory follow-ups of prematurely born children, Bodgan et al. also found that former very premature infants were prone to long-term respiratory sequelae [23]. The reasons are mainly related to impaired lung development due to dysmaturity, infectious agents, mechanical ventilation, and deficient control of breathing. The consequences are an increased risk of viral infections, recurrent wheezing, asthma, and a decrease in airway flow. Therefore, the authors suggest that very prematurely born children should be followed in an organized fashion to undertake preventive strategies to improve these vulnerable infants’ prognoses [23]. Early preventive strategies might impact their long-term lung health, lowering the risk of premature adult mortality [24]. Systematic follow-up organizations are now well developed in many countries via perinatal and follow-up networks [25]. Likewise, in a systematic review of studies on the outcomes of teenagers with BPD, Carrega et al. evaluated 31 studies up to March 2023 [26]. They found that teenagers with a history of BPD presented more respiratory symptoms, such as wheezing and respiratory exacerbations, with an impairment in pulmonary function. There was a higher risk for special education needs, but the quality of life seemed similar to that of non-BPD adolescents. The data from their review are consistent with the results of our study.

In a study about the health-related quality of life (HRQoL) in teenager survivors of very premature birth, Bozzetto et al. compared 27 BPD patients, 27 asthmatic patients, and 27 healthy controls using the Short-Form 36 questionnaire [27,28]. Despite impaired lung function according to their spirometry, the BPD patients had a similar quality of life to the controls, which was better than the asthmatic patients. In a study on psychological functioning and HRQoL in adulthood after preterm birth, Dalziel et al. found, in 126 young adults born prematurely compared to 66 born at term, that adults born preterm had an HRQoL consistent with those who were born at term [29]. However, BPD may be associated with impaired executive functioning [30], and the HRQoL may actually be related to the level of prematurity, brain lesions, and the presence of neurosensory or cognitive limitations [31]. This could explain some of the discrepancies observed in the literature. In addition, although the patients themselves may not recognize a lower HRQoL, impaired functioning is often associated with parents reporting a lower HRQoL. Finally, in a longitudinal study, Berdal et al. showed that VPN-born adults reported a lower HRQoL than term-born controls at 32 years of age, with a significant decline from 20 to 32 years of age, especially for VPN-born individuals with disabilities [32]. Therefore, one must be careful in evaluating the HRQoL early in life and stay aware of difficulties appearing later in life, which may benefit from appropriate support on a long-term basis.

It is worth noting that more than 40% of the infants involved in the CURDYS trial were initially treated by probabilistic antibiotics because of a high suspicion of materno-fetal infection. In addition, about 90% of them presented late-onset sepsis when the overall rate of infection ranged from 14% to 24% in the participating centers for infants below 33 weeks of gestation. This is consistent with the results of Paananen et al. [33], who studied 128 very premature infants and found that infants with inflammation and high concentrations of inflammatory cytokines were at high risk of BPD. Thus, in addition to the neurological impairment associated with BPD [34], it is now recognized that the exposition of the immature brain to inflammation contributes to significant cerebral injury and adversely affects brain development, leading to subsequent neurological impairment [35]. The role of inflammation in the development of BPD may also be responsible for part of the poor neurodevelopment outcomes of the infants who had presented severe RDS and subsequent BPD in the neonatal period. In a study of 98 VPN children who had BPD compared to 75 VPNs without BPD and 99 healthy term-born children, Short et al. evaluated the cognitive and academic consequences of BPD at 8 years old [36]. As observed in our study, they showed that 54% vs. 37% of VPNs without BPD and 25% of term-born infants were more likely to require special education support. They showed the same rate as in our study, with 20% of BPD children presenting a full-scale IQ < 70, compared to 11% of VPNs without BDP and 3% of term-born infants. They observed, as in other studies, a significant influence of the maternal education level. In our study, there were no differences in the maternal or parental education levels between the two groups. Overall, the authors conclude that BPD and a long duration of oxygen had long-term adverse effects on cognitive and academic achievement. These findings highlight the need for learning, behavior, and development support in BPD children to improve their learning abilities and attenuate their school difficulties.

### 4.1. Limitations of the Study

Our study has limitations. The multicenter design may lead to some heterogeneity in the PFT method among centers despite a standardized protocol. However, the use of the z-score for all children may improve the generalization of the results. Likewise, because of the heterogeneity among the centers, we were not able to reliably record the medications used by the children during the study period besides their asthma treatment. This does not modify the message of the study, but it emphasizes the burden on health resource utilization and the overall cost of the infants’ care. In addition to the clinical interest in surfactant treatment in the neonatal period, surfactant administration could be cost-effective in the long term, but this warrants further study.

Secondly, although we were able to follow 80% of the eligible survivors, the number of studied children was rather low, and some results may lack power. Of note, six children were not able to perform PFT and were not able to pass the WISC-IV evaluation because of insufficient understanding abilities. This worsens the actual results observed and reported. However, the children were equally distributed in both groups and an impact on the overall message is unlikely.

Finally, using the forced oscillation technique (FOT), an alternative method for measuring lung function during tidal breathing, and thus, requiring minimal patient cooperation, would have resulted in increased PFT performance in the children and brought additional information. This method is widely used to explore alterations in respiratory mechanics localized to more peripheral airways, for which spirometry was shown to be less reliable [37]. Indeed, our results of the mid-expiratory flows (MMEFs) do not show a significant difference between the two groups due to the great variability in this parameter. However, the MMEF z-score values in our control group are similar to those found in the study by Simpson et al. [38], who analyzed the lung structure and function of mid-childhood survivors of very preterm birth (≤32 weeks of gestation) and reported worse peripheral respiratory mechanics than in healthy children when using the FOT. Altered lung structure and development, together with the suggested inflammatory process in preterm lungs, has negative effects on airway function and strongly indicates possible small airway involvement. Several monocentric studies suggest that all FOT outcomes, but especially those related to the elastic properties of the respiratory system, may display some ability to detect lung disease resulting from preterm birth [39]. Using FOT might prove useful in the evaluation of repeated surfactant instillation on lung function, however, the need for harmonization and the lack of FOT availability in some of the 13 investigation centers made it impossible to use this technique for this multicenter study.

### 4.2. Future perspectives

The results of the CURDYS trial are consistent with new approaches of using surfactants not only for “physiologic” replacement, soon after birth, but repeated as a medication for infants with severe prolonged respiratory distress. Other strategies of treatment using surfactants as a vehicle showed promising results. After verifying the biochemical properties, Yeh et al. [40] showed that 128 infants treated with surfactants mixed with budesonide showed a significant reduction in the risk of BDP or death when compared to 134 controls (OR 0.58; 95% CI 0.44–0.77). Their study is very interesting, as we observed a significant morbid association between respiratory distress and inflammation. Finally, the approach of using human umbilical cord-derived mesenchymal stem cells combined with surfactants is under investigation and has shown promising results in animal studies [41].

## 5. Conclusions

Beyond its importance as a physiological replacement soon after birth, the use of curative repeated surfactant therapy in infants presenting severe prolonged respiratory distress improves lung function and lowers the risk of asthma on a long-term basis. However, it does not improve poor neurodevelopment, which might also be related to inflammation. The implications of this study are that repeated surfactant therapy in the neonatal period may be an option to alleviate early lung injury, allowing for long-term lung health improvement in association with early preventive strategies, such as strict smoking exclusion and reasonable physical exercise. The new approaches to treatments combining surfactants and budesonide are promising.

## Figures and Tables

**Table 1:** Characteristics of the population studied.

Characteristics ^1^	Surfactant-Treated Children	Controls
Gestational age, weeks	26.1 ± 1.3	26.1 ± 1.4
Post-natal age at evaluation, y	7.17 ± 0.29	7.15 ± 0.26
Maternal education level (%):		
. Elementary school	2.8	5.4
. High school	30.5	24.4
. Baccalaureate	25.0	35.1
. College	41.7	35.1
Paternal education level (%):		
. Elementary school	2.9	5.5
. High school	37.1	36.1
. Baccalaureate	22.9	27.8
. College	37.1	30.6
Gender (% male)	56	59
Weight, kg (z-score)	22.2 ± 5.1 (−0.259 ± 1.270)	21.4 ± 5.3 (−0.465 ± 1.292)
Height, m (z-score)	1.19 ± 0.07 (−0.525 ± 1.222)	1.18 ± 0.06 (−0.722 ± 1.031)
Head circumference, cm	51.6 ± 1.7	50.8 ± 1.6
Cerebral palsy, %	5.0	2.8
Sleeping problems, %	10.0	13.4
Deafness, %	12.5	11.1
Visual problems % ^2^	46.3	52.8

^1^ No differences were significant; ^2^ including glasses.

**Table 2:** Parents’ responses to the auto-questionnaire.

Domain Scores (Min–Max) ^1^	Surfactant-Treated Children	Controls
Wheezing any time, %	63	76
Diurnal symptoms (0–16) ^2^	7 (0; 12)	7.5 (0; 12)
Night symptoms (0–16)	1 (0; 2)	1 (0.25; 2)
Impact on child’s QoL (0–16)	2 (0; 5)	1 (0; 4)
Impact on family’s QoL (0–16)	1 (0; 3)	1 (0; 0.3)
Global score (0–64)	8 (0; 17)	9 (0; 19)

^1^ Median (IQR); no differences were significant. ^2^ Minimum–maximum values. QoL: quality of life.

**Table 3:** Pulmonary function testing comparison between the 2 groups.

Values (Mean ± SD)	Surfactant-Treated Children	Controls	*p*
**FEV1 ^1^**	**1.188 ± 0.169**	**1.080 ± 0.243**	**0.032**
FEV1 z-score	−0.803 ± 1.045	−1.446 ± 1.270	0.061
**FEV1 post β+ ^2^**	**1.244 ± 0.183**	**1.091 ± 0.209**	**0.030**
FEV1 post β+ z-score	−0.525 ± 1.003	−1.342 ± 1.133	0.056
**FVC ^3^**	**1.402 ± 0.217**	**1.265 ± 0.267**	**0.049**
**FVC z-score**	**−0.406 ± 0.217**	**−1.141 ± 1.217**	**0.022**
FVC post β+	1.452 ± 0.237	1.279 ± 0.264	0.054
**FVC post β+ z-score**	**−0.241 ± 0.907**	**−1.020 ± 1.207**	**0.045**
MMEF ^4^	1.287 ± 0.412	1.153 ± 0.441	0.253
MMEF z-score	−1.079 ± 1.153	−1.446 ± 1.190	0.268
MMEF post β+	1.332 ± 0.335	1.226 ± 0.351	0.768
MMEF post β+ z-score	−0.877 ± 0.938	−1.189 ± 1.032	0.474
FRC ^5^	1.165 ±0.280	1.208 ± 0.304	0.513
TLC ^6^	2.179 ± 0.358	2.137 ± 0.343	0.974
VC ^7^	1.385 ± 0.210	1.270 ± 0.293	0.142
sRaw ^8^	10.233 ± 3.564	11.604 ± 8.945	0.312
sRaw post β+	7.459 ± 2.505	8.666 ± 5.079	0.760

^1^ FEV1 (L/s): forced expiratory volume in one second; ^2^ β+: betamimetics; ^3^ FVC (L): forced vital capacity; ^4^ MMEF (L/s) maximum mid-expiratory flow^5^ FRC (L): functional residual capacity; ^6^ TLC (L): total lung capacity; ^7^ VC (L): vital capacity; ^8^ sRaw: specific airway resistance.

**Table 4:** WISC-IV subtest evaluations.

Subtests	Surfactant-Treated Children	Controls
Verbal Comprehension Index	10 (9–12)	10 (8–11.3)
Perceptual Reasoning Index	7 (6–10.3)	6 (5–9)
Working Memory Index	9 (8–10)	8 (6.8–10)
Processing Speed Index	9 (6–10)	9 (5–10)

Median (IQR); no differences were significant.

## Data Availability

The data presented in this study are available upon request from the corresponding author. The data are not publicly available due to the absence of an accessible repository and a change in hospital responsibility in the middle of the study (a fusion between the Maternity Regionale University Hospital and the CHRU of Nancy occurred in 2014).
